# How do episodic and semantic memory contribute to episodic foresight in young children?

**DOI:** 10.3389/fpsyg.2014.00732

**Published:** 2014-07-08

**Authors:** Gema Martin-Ordas, Cristina M. Atance, Julian S. Caza

**Affiliations:** ^1^Cognitive Zoology, Department of Cognitive Science, Lund UniversityLund, Sweden; ^2^School of Psychology, University of OttawaOttawa, ON, Canada

**Keywords:** episodic memory, semantic memory, episodic foresight, children, developmental psychology

## Abstract

Humans are able to transcend the present and mentally travel to another time, place, or perspective. Mentally projecting ourselves backwards (i.e., *episodic memory*) or forwards (i.e., *episodic foresight*) in time are crucial characteristics of the human memory system. Indeed, over the past few years, episodic memory has been argued to be involved both in our capacity to retrieve our personal past experiences and in our ability to imagine and foresee future scenarios. However, recent theory and findings suggest that semantic memory also plays a significant role in imagining future scenarios. We draw on Tulving’s definition of episodic and semantic memory to provide a critical analysis of their role in episodic foresight tasks described in the developmental literature. We conclude by suggesting future directions of research that could further our understanding of how both episodic memory and semantic memory are intimately connected to episodic foresight.

## INTRODUCTION

The human mind often wanders forward in time to contemplate what the future might be like (e.g., our next conference talk) and backwards in time to re-experience personal past events, or what is referred to as *episodic memory* (Tulving, 1983, 2005). The ability to mentally travel forward in time has been referred to as: *episodic future thinking* (Atance and O’Neill, 2001), *prospection* (Buckner and Carroll, 2007; Gilbert and Wilson, 2007), *simulation* (Tulving, 1985; Schacter and Addis, 2007; Atance, 2008; Schacter et al., 2008), *projection* (Okuda et al., 2003), *mental time travel into the future* (Suddendorf and Corballis, 1997; Klein, 2013), *episodic simulation of future events* (Schacter et al., 2007), *episodic foresight* (Suddendorf, 2010) and, more recently, *future-oriented cognition* (Osvath and Martin-Ordas, in press). Although these different terms refer to different degrees or types of future cognition, they all share the idea that future thinking entails imagining a personal future event. In this article we use the term *episodic foresight* to describe this particular ability.

The adaptive function of episodic memory is suggested to lie not only in record-keeping of the past, but in what it can offer to present and future fitness (Suddendorf and Corballis, 1997, 2007; Dudai and Carruthers, 2005; Tulving, 2005; Buckner and Carroll, 2007; Schacter et al., 2007; Klein, 2013). For example, when I try to imagine my next job interview, I might retrieve memories of previous job interviews so I can avoid certain (embarrassing) situations. In fact, from an evolutionary perspective, imagining and planning for one’s personal future based on memories of past events, and planning for future events that do not depend on current needs is argued to provide an important selective advantage (Suddendorf and Corballis, 2007; Klein, 2013).

Research in the past few years has shown that episodic memory and episodic foresight share numerous cognitive and neural resources (e.g., Williams et al., 1996; Klein et al., 2002; Okuda et al., 2003; Tulving, 2005; Addis et al., 2007; Hassabis et al., 2007; Szpunar et al., 2007). Importantly, however, both theory and data about the important role of semantic memory in episodic foresight have also appeared (e.g., Irish and Piguet, 2013), with some studies showing that semantic memory might be sufficient for episodic foresight (Klein et al., 2002). For example, a group of patients with episodic amnesia, but normal semantic knowledge, were able to succeed in a future decision-making (e.g., delay discounting) task even though they could not imagine themselves in future events (Kwan et al., 2013).

The extent to which both semantic and episodic memory may be implicated in episodic foresight is interesting from a developmental perspective given that a well-accepted (but by no means uncontroversial) argument is that children develop semantic memory prior to episodic memory (e.g., Wheeler et al., 1997). If so, then children’s performance on tasks of episodic foresight may have important implications about the respective roles of episodic and semantic memory. For example, a 3-year-old who is unable to think about the future could conceivably be seen as having a limitation in episodic memory, rather than semantic memory, since the latter could be argued to already be fairly well-developed. In contrast, if a 3-year-old performs well on a task of episodic foresight, then it is arguable that semantic memory is sufficient to succeed. As we will discuss shortly, research (including developmental) has shown an important link between episodic memory and episodic foresight. However, the role of semantic memory has received less attention yet, presumably, it, too, plays a significant role in thinking about the future.

The major goal of this paper is to analyze the relation between semantic memory, episodic memory, and episodic foresight in the context of developmental research. We first lay the groundwork for an analysis about how these two types of memory may contribute to episodic foresight in children by briefly reviewing contrasting theories about the distinctiveness of episodic and semantic memory in adults. We then provide an overview of the research on episodic foresight in young children, paying careful attention to age differences on these tasks because these can inform us about potential memory processes involved. An important point that we then build upon is the idea that in, certain contexts, semantic memory is an important driver of episodic foresight. With this point in mind we re-visit tasks of episodic foresight in children with the goal of determining the extent to which both semantic and episodic memory are necessary correlates. We then conclude by suggesting possible avenues of future research with children that will further help to elucidate the relative roles of semantic and episodic memory in episodic foresight.

## SEMANTIC AND EPISODIC MEMORY

### GENERAL OVERVIEW

Tulving (1972) introduced one of the most influential distinctions in the study of memory: semantic memory and episodic memory. Semantic memory was originally defined as our database of knowledge about the world. In contrast, episodic memory was considered “an information processing system that (a) receives and stores information about temporally dated episodes or events, and about temporal–spatial relations among these events, (b) retains various aspects of this information, and (c) upon instructions transmits specific retained information to other systems, including those responsible for translating this information into behavior and conscious awareness” (Tulving, 1972, p. 385). Thus, when we state that “winters in Canada are cold” we are drawing on semantic memory; however, when we remember walking on the snowy streets of Ottawa a couple of winters ago, we are drawing on episodic memory.

Tulving (1983, 2005) gradually refined the defining features of these memory systems. He argued that episodic memory makes possible *mental time travel* through subjective time, from the present to the past, thus allowing one to re-experience past events (Tulving, 1983). Another critical addition to his original model was that a certain form of consciousness – *autonoetic* (“self knowing”) *awareness* – was a necessary indicator of episodic memories. Tulving (1983,2005) defined this type of consciousness as a type of first-person phenomenological experience, which is based on previous experiences, is detached from current perceptions, and provides its owner with the feeling that the experience belongs to his/her temporal self. This is in contrast to the semantic system, where knowledge about facts is not subjectively tied to past experiences but simply involves awareness of familiarity of knowing: i.e., *noetic* consciousness.

### THE BLURRY LINE BETWEEN SEMANTIC AND EPISODIC MEMORY

The distinction between episodic and semantic memory was a central feature of Tulving’s original conceptualization with the most convincing evidence for such a distinction coming from neuropsychological studies (Kapur, 1999; Conway and Fthenaki, 2000; Wheeler and McMillan, 2001). For example, patients with medial temporal lobe lesions have been shown to lose the ability to use episodic memory while retaining other classes of memory, including semantic memory (Vargha-Khadem et al., 1997; Hirano and Noguchi, 1998; Gadian et al., 2000). Similarly, patients with semantic dementia have severe semantic memory loss, while their episodic memory is relatively spared (Snowden et al., 1994; Graham et al., 2003; McKinnon et al., 2006). Thus, dissociations between these two memory systems suggest their independence.

However, evidence for a clear-cut division between these two types of memories is somewhat contentious (Squire et al., 2004; Tulving, 2005). This is reflected in the following example. When I remember my last winter in Ottawa (e.g., my apartment, the snow, wearing warm clothes, the Rideau canal being frozen), I am retrieving an episodic memory; however, facts such as “winters in Canada are usually associated with snow” reflect semantic knowledge and provide the backdrop upon which the episodic details are integrated. This example nicely illustrates that semantic memory penetrates episodic memory (Balota and Coane, 2008). Neuropsychological research has also demonstrated such an interdependent relation between episodic and semantic memories (see Greenberg and Verfaellie, 2010, for a review).

Similarly, repetition of events across time tends to transform episodic memories into semantic memories (e.g., memories from my time living in Canada). Interestingly, this kind of memory does not seem to have a specific temporal component, be associated with any specific event, nor involve autonoetic conscious experience (i.e., I might remember several events associated with that period of my life). Thus, one might be tempted to not consider them as reflecting episodic memory. Note, though, that these memories can be rich in other contextual details. In fact, if I were asked to remember and describe my apartment in Ottawa, I could probably provide precise details about its location, the color of the walls, or its spatial arrangement. Importantly, these features are bound to the spatial context, in which they were experienced. Such contextual binding is crucial for memories to be considered episodic (Chalfonte and Johnson, 1996; Eichenbaum, 1997; Newcombe et al., 2007). Thus, the recollection of specific features of those repeated events suggests that these memories have not been decontextualized and, therefore, these memories cannot be drawn exclusively from semantic memory. Memories like these are not explicitly addressed in the classic episodic-semantic model. However, they fit with Neisser’s (1981) idea of merging of memories for past events into one representative event, Barsalou’s (1988) concept of summarized or extended events, or Conway’s (2001) “general events” level of autobiographical knowledge and thus provide an example of how episodic and semantic features are intertwined.

Because of such arguments highlighting the overlap between episodic and semantic memory, Tulving revisited his original model and re-defined the relation between these two forms of memory. In his new (hierarchical) model (termed SPI-Serial encoding, Parallel storage, and Independent retrieval), episodic memory became a subsystem of semantic memory and was considered to be dependent upon the integrity of semantic knowledge (Tulving, 1995). That is, information can be encoded into semantic memory independently of episodic memory. However, information can only be encoded into episodic memory through semantic memory. Nonetheless, Tulving’s SPI model has been empirically questioned (e.g., Squire and Zola, 1998; Graham et al., 2000). For example, Squire and Zola have argued that the encoding and retrieval of semantic memory depends on the acquisition of the episode in which such information was experienced.

These different strands of evidence highlight that the contents of episodic memory invariably involve semantic representations and that there is clear inter-dependency between these two systems. As such, it may follow that episodic future thoughts also contain semantic elements. We explore this possibility next from a developmental perspective.

## THE DEVELOPMENT OF FUTURE THINKING/FUTURE-ORIENTED COGNITION

In this section we provide a brief review of the history of research on episodic foresight in young children. We then shift to considering the research in this area that has shown a link between children’s memory for the past and their thought about the future.

### A BRIEF OVERVIEW OF RESEARCH ON EPISODIC FORESIGHT

Although the study of children’s episodic foresight is relatively new, the extent to which children think about the future has not been completely overlooked by developmental psychologists. In addition, the claim that thought about the future is based on our memories of the past was also made by researchers prior to the 1990s (e.g., Harner, 1982; Nelson, 1989; Weist, 1989; Friedman, 1990). Some of the earliest research on children’s conception of the future was conducted in the area of language, with a number of researchers reporting that around 2 years of age children begin to verbally reference both the past and the future (e.g., Sachs, 1983; Eisenberg, 1985; Nelson, 1989). Although children’s early talk about the future appears to be built upon their memories of the past (e.g., Nelson, 1989), it is important to note that novel details are also incorporated into these accounts thus supporting the claim that factors other than memory play an important role (Atance and Martin-Ordas, 2014; though such factors will not be the focus of our paper). In what follows we outline work on episodic foresight dividing it into the following two general categories: (1) verbal tasks, and (2) non-verbal tasks.

#### Verbal tasks

A number of researchers have studied children’s episodic foresight by simply asking them to talk about the future. For example, Hudson et al. (1995) asked children between the ages of 3 and 5 to report both plans and scripts for future events such as going to the beach and going grocery-shopping. They found that whereas children’s “script” reports did not improve significantly between ages 3 and 5, their plans for these events did. Interestingly, Atance (2008) has argued that reporting scripts may rely to a greater extent on semantic memory, whereas reporting plans (with their inherent focus on what “I” will do, rather than the generic “what happens”) relies more heavily on the episodic system. Thus, conceivably, the younger children whose episodic memory is not as well developed as the older children may have struggled with the “plan” condition but performed relatively well (i.e., no differently than the older children) in the script condition because they, too, were able to rely on their semantic memory.

Another verbal approach has simply been to ask children to report what they will do “tomorrow.” In doing so, Busby and Suddendorf (2005) and Suddendorf (2010) found that 4- and 5-year-olds tended to report future events that their parents deemed “accurate,” whereas 3-year-olds tended not to. Interestingly, 3-year-olds did not seem to be limited by their verbal ability but, rather, by their inability to predict an event that might plausibly occur in their personal futures. Of course, a not mutually exclusive possibility is that older children succeeded by retrieving a specific past episode and merely projecting it into the future (as opposed to thinking about the future, *per se*). The fact that parents were asked to verify the accuracy of children’s reports reduces – but does not eliminate – this possibility. Nonetheless, we return to this study later in the paper because its findings have implications for the relative roles that semantic and episodic processes may play in children’s ability to talk about the future.

In contrast to the significant age differences detected between 3 and 5 using the “tomorrow” method, more recent studies have found that 3-year-olds are more skilled at talking about future events than was reported by Busby and Suddendorf (2005) and Suddendorf (2010). For example, Quon and Atance (2010) asked children to respond to questions about *specific* events that would occur tomorrow (e.g., “What are you going to have for breakfast tomorrow morning?” or “What are you going to do the next time you go to the park?”). Overall, approximately 63% of 3-year-olds’ reports were deemed accurate by their parents (vs. approximately 30% in Busby and Suddendorf’s 2005, study). Although Quon and Atance (2010) also found that performance improved with age, children seemed to perform better when asked about a specific future event vs. the future more globally (e.g., “tomorrow”). This finding further suggests that children have particular difficulty *generating* a plausible event in response to a general temporal cue like “tomorrow.” However, when asked about a more specific event (e.g., breakfast), generating the necessary information appears to be easier. If this line of reasoning is correct, then one hypothesis is that children should be able to describe events even more accurately were the event in question generated for them. And, indeed, findings by Hayne et al. (2011) support this claim.

Rather than asking children, themselves, to generate future events, Hayne et al. (2011) asked parents to produce one event that would likely happen to them and their child later that day, and a second event that they would likely experience the next day. Children were then interviewed about the future events provided by their parents. After the testing session, each parent was sent a transcript of what his/her child had said about the future events and asked to rate his/her child’s accuracy. Hayne et al. found that both 3- and 5-year-olds provided information about the future events and, interestingly, there was no age difference in accuracy between these two age groups (though 5-year-olds provided more information per event than 3-year-olds).

#### Non-verbal tasks

A number of researchers have also used methods that could be considered more “non-verbal” to tap into children’s episodic foresight. For example, Atance and Meltzoff (2005) showed 3-, 4-, and 5-year-olds photographs that depicted such scenarios as walking beside a waterfall, walking through a desert, and hiking up a mountain. Children were asked to pretend that they would visit these locations and to select one of three items to bring with them. The correct item (e.g., raincoat) could be used to address a future physiological state (e.g., getting wet), while the other two distracter items could not. Each of the three age groups chose the correct items at a rate higher than would be expected by chance (74, 91, 97, for the 3-, 4-, and 5-year-olds, respectively); though, overall, 4- and 5-year-olds performed significantly better than the 3-year-olds.

Using another type of “item-selection” task, Russell et al. (2010) taught 3-, 4-, and 5-year-olds to play a game in which the child and the experimenter stood on either side of a table with a goal at each end and a ball to blow into it. Materials for this game of “blow football” included non-essential items that were thematically related to the game (e.g., cardboard referee, team badge), as well as one essential item: a straw with which to blow the football. To play the game from the experimenter’s side (the “blue” side), however, children needed a box to stand on. Children first played the game with the experimenter from the “red” side and were then told that they would be returning the next day to play the game from the blue side. Children were then asked to select two items (from an array of six) that would be needed for “tomorrow.” Only 5-year-olds selected the correct item pair (box and straw) more often than would be expected by chance.

Finally, Suddendorf and Busby (2005) and Suddendorf et al. (2011) have specifically targeted the extent to which children’s current actions/choices are driven by the anticipation of future events or needs. In fact, Suddendorf and Corballis (2007) proposed one of the most commonly used criteria of episodic foresight that has been used by both developmental and comparative psychologists. They suggested that having the future in mind should, at minimum, provide *flexibility* to the owner of such a behavior. In addition, one should rely on a single *unique experience* (i.e., one trial experience) and there should not be any *cue/stimuli* in the present environment that could trigger such a response.

Based on these criteria, Suddendorf and Busby (2005), Suddendorf et al. (2011), and Redshaw and Suddendorf (2013) have developed tasks in which children are presented with a “problem” in one room (e.g., a box with no key to open it) and then experience a brief delay (e.g., 15 min) in another room. After this brief delay, children are told that they will return to the first room and are presented with several options; one of which (i.e., a key) can be used to open the box and retrieve a reward (e.g., a sticker). This experiment fulfills at least two of Suddendorf and Corballis’ (2007) criteria because children’s performance is based on a single unique experience and, at the time they are asked to make a choice, there is no (visible) task that can trigger the selection of the tool. In these studies, 3-year-olds tend not to select the correct item at a rate higher than chance, whereas 4-year-olds do. However, if 3-year-olds are tested with no delay between the presentation of the problem and the opportunity to select an item (with their back turned to the problem so that they cannot see it), their performance is significantly above chance (e.g., Suddendorf et al., 2011). Although we will return to this issue later in the paper, the need for children to remember past information (e.g., shape of a keyhole) is a necessary requirement for task success.

In sum, both verbal and non-verbal measures of episodic foresight tend to show age-related changes between ages 3 and 5. However, it is important to note that there are exceptions to this general pattern. For example, Hayne et al. (2011) did not find age differences and Russell et al. (2010) found that only 5-year-olds succeeded on their task. These age differences are consistent with the idea that the extent to which memory (episodic or semantic) is involved in task success may vary as a function of the specific episodic foresight task.

In the next section, we discuss some of these developmental studies in greater detail because they are relevant to the argument that children’s memory for the past is related to their thought about the future. More specifically, if semantic and/or episodic memory are indeed required for children’s episodic foresight, then the extent to which children remember the past should be related to their capacity to think about the future. Detecting this relation thus sets the stage for the more specific argument that both episodic *and* semantic memory are critical for children’s episodic foresight.

### LINKS BETWEEN MEMORY FOR THE PAST AND THOUGHT ABOUT THE FUTURE

#### Verbal tasks

In addition to asking children to report events that they would do “tomorrow,” Busby and Suddendorf (2005) asked them to report two things that had happened “yesterday.” As with children’s responses to the “tomorrow” question, parents were asked to state whether these responses were correct/incorrect. If memory is indeed critical for episodic foresight, then children should perform similarly on the yesterday and tomorrow questions. And, indeed, a majority of 4- and 5-year-olds (56 and 75%, respectively) accurately reported events from the past and the future, whereas only a minority of 3-year-olds did (25%). In a subsequent study using similar methods, Suddendorf (2010) again reported that children who answered the “yesterday” question correctly were also likely to do so for the “tomorrow” question. In addition, there was a significant correlation between the number of accurate responses generated to the yesterday and tomorrow questions. Hayne et al. (2011) also showed positive significant correlations between the amount of information provided about past and future events.

#### Non-verbal tasks

Several of the non-verbal tasks that we outlined earlier also speak to the memory-foresight link. More specifically, recent studies using variations of Suddendorf et al.’s (2011) paradigm indicate that children’s failure to select the correct item (e.g., key) to solve a past problem (e.g., locked box) is mostly driven by children’s inability to remember the past problem (or features of it; e.g., Scarf et al., 2013; Atance and Sommerville, 2014). For example, Atance and Sommerville (2014) gave children a series of tasks in which they encountered a problem in one room (e.g., a glass of juice glued to a tray with no straw with which to drink it) and then, several minutes later in another room, were given various items with which to solve the problem. One of these items (i.e., a straw) was the correct choice. In addition to asking children to select an item to bring back with them to the first room, children were afterwards asked to state what had been on the table (the location where the “problem” had been placed) in the first room. Atance and Sommerville (2014) found that when they controlled for 3-, 4-, and 5-year-olds’ responses to this *memory question,* there were no significant age-related increases in children’s correct item choices (e.g., selecting the straw in anticipation of drinking the juice) – the variable ostensibly measuring episodic foresight. This suggests that task performance was primarily driven by children’s memory for the past problem, and not necessarily by a “future thinking” component. Moreover (and perhaps as expected), children’s memory for the problem and their correct item choices were significantly related.

Taken together, these studies are consistent with the claim that children’s ability to think about the future (or, “episodic foresight”) is related to their memory for the past. However, what particular aspect of the past and, hence, what form of memory can be argued to be critical to task success? Although the assumption is that it is episodic memory, to what extent might semantic memory also be necessary? We next explore how episodic foresight may be driven by *both* episodic and semantic memory.

## THE ROLE OF EPISODIC MEMORY AND SEMANTIC MEMORY IN EPISODIC FORESIGHT

### IS EPISODIC MEMORY NECESSARY FOR EPISODIC FORESIGHT?

Atance and O’Neill (2001,2005) suggested that Tulving’s “episodic-semantic” distinction could also be applied to future thinking. Thus, episodic foresight (or, “episodic future thinking” as these authors termed it) was described as the capacity to mentally project oneself into the future to “pre-experience” a spatio-temporally specific event (e.g., imagining my next visit to Ottawa). In contrast, semantic future thinking was originally conceived as the capacity to think about facts and context-free general conceptual knowledge (e.g., there is going to be an election next year; Atance and O’Neill, 2001, 2005; Klein et al., 2002).

As mentioned earlier, the idea that episodic memory is crucial for imagining future events is widespread in episodic foresight research (Schacter and Addis, 2007; Suddendorf and Corballis, 2007; Szpunar and McDermott, 2008). More specifically, episodic memory is argued to allow for the extraction and combination of stored information to create new events. In addition, authors have argued that past and future events draw on similar information and rely on common underlying processes (Schacter and Addis, 2007; Suddendorf and Corballis, 2007; Klein, 2013). Thus, past experiences are used to anticipate possible future events and this is argued to have an important adaptive value. This hypothesis has been confirmed in a number of neuropsychological studies in which patients with impaired episodic memory fail not only at retrieving episodic memories but also at imagining personal future events (Tulving, 1985; Hassabis et al., 2007; Addis et al., 2009; Gamboz et al., 2010; Kwan et al., 2010; Race et al., 2011).

It is important to note, however, that one can retrieve episodic information to anticipate a future situation without necessarily mentally projecting the s*elf* into this situation (Pillemer, 2003; Szpunar, 2010; Osvath and Martin-Ordas, in press). For instance, as Osvath and Martin-Ordas (in press) mention remembering the last time I forgot my keys and got locked out of my apartment does not imply that every time I take my keys I do so because I project myself into the future event of having to call the locksmith. Note though that the behavioral outcome in both situations (i.e., imagining the specific details of the future event vs. only anticipating the negative consequences of forgetting my keys) is the same (i.e., I take my keys). This idea resembles the “mnemonic associative theory” already suggested by Cheke and Clayton (2010), in which it is argued that associate learning and future-oriented cognition might suffice for future thinking (i.e., “known future” as termed by Klein, 2013).

So far we have described evidence supporting the idea that episodic memories are the basis for episodic foresight. However, recent research has brought into question the exclusive role of episodic memory in episodic foresight. In the following section we review evidence to this effect.

### THE ROLE OF SEMANTIC MEMORY IN EPISODIC FORESIGHT

The contribution of semantic memory to episodic foresight has received much less empirical attention than the link between episodic memory and episodic foresight (but see; Szpunar, 2010; D’Argembeau et al., 2011; Martin-Ordas et al., 2012; Klein, 2013). Most of the evidence for the importance of semantic memory in episodic foresight has come from neuropsychological research (e.g., Duval et al., 2012; Irish et al., 2012; Irish and Piguet, 2013). For example, Irish et al. (2012) explored the contribution of semantic memory to episodic foresight in semantic dementia patients (i.e., semantic memory impaired, episodic memory spared) and Alzheimer’s disease patients (i.e., semantic memory spared, episodic memory impaired). The results were striking: Whereas Alzheimer’s patients showed problems retrieving episodic memories and imagining personal future events, semantic dementia patients remembered past events but were impaired in episodic foresight. Importantly these results suggest that semantic memory may be necessary for the construction of future events. Similarly, Maguire et al. (2010) have shown that patients with hippocampal damage have an intact ability to construct imagined experiences despite deficits in the episodic memory system.

Taken together, these results pose difficulty for the idea that the capacity for imagining new experiences is exclusively dependent on *episodic* details from past events. With these ideas in mind, we re-evaluate the work that has been done on children’s episodic foresight to see if it can provide us with insights about how both semantic and episodic memories allow children to construct future events.

### CRITICAL RE-EVALUATION OF THE DEVELOPMENTAL WORK ON EPISODIC FORESIGHT

#### Verbal tasks

Recall that one difficulty that 3-year-olds appeared to have when predicting events that would happen “tomorrow” was generating events that their parents judged would actually happen the next day. In other words, it was not the generation of an event in the global sense that was difficult but, rather, the generation of an event that would actually involve/happen to *them.* One intriguing interpretation of these findings is that, initially, younger children generate “a future” based predominantly on semantic knowledge about the world. More specifically, children may be more apt to generate well-known “scripts” or “routines.” For example, children may predict birthday parties, going to the restaurant, going to the park, etc. without paying careful attention to whether this event is likely to occur in their immediate future (i.e., *known future*). If so, parents will report that this event is unlikely to occur the next day. In contrast, older children may be better at thinking more accurately about the specific events that they will engage in the next day. Thus, rather than generating *a* future, as might be the case with younger children, they are better at generating their *own* future (see Klein, 2013 for a similar argument). This might imply that older children are relying on semantic memory (e.g., they can apply script knowledge about the event) but also that they may be able to imagine their own future and filter out, or “screen,” those events that are not likely to occur to them the next day. It is possible that this filtering process is a function of the episodic system. Indeed, Atance and O’Neill (2005) argue that one key factor that differentiates episodic foresight from related processes (e.g., imagination) is the notion of constraints. For example, if one has a broken leg, it is possible to imagine the event of going skiing (with this process likely drawing largely on semantic knowledge about skiing) yet, one cannot realistically project into this event because of the constraint of having a broken leg.

If our line of reasoning is correct, this would imply that providing younger children with the event in question (past or future) would then allow them to elaborate upon it (but they would nevertheless have difficulty generating it themselves). This argument is consistent with Hayne et al. (2011) who found that both 3- and 5-year-olds could provide details about past and future events and, importantly, that there were no age differences in this respect. This is in contrast to Busby and Suddendorf’s (2005) study in which 4- and 5-year-olds performed better than 3-year-olds. However, the important difference between these two studies is that, in the latter, children were asked to generate events they did yesterday or would do tomorrow whereas, in the former, the experimenter provided children (from parents’ accounts) with the events that had either happened/would happen in their lives and asked them to elaborate on them. In sum, whereas in Busby and Suddendorf children needed to generate the events in question, in Hayne et al. children were provided with a clear cue upon which they could elaborate their reports. What this might imply is that the episodic information is available to both younger and older children, but the way in which this information is accessed differs across ages. Whereas older children might be able to both generate their own cues and use external cues, younger children might only be able to access past memories when provided with external cues.

Conway (2005) has already introduced the distinction between *generative* retrieval and *direct* retrieval: whereas *generative* retrieval requires elaborating upon one’s own cue to retrieve a memory (e.g., when asked about your last conference, you need to elaborate the necessary cues to retrieve events associated with it), in *direct* retrieval, the cue is provided by the context (e.g., a certain smell can bring to mind several memories associated with a life period). Hayne et al.’s (2011) results are relevant to episodic foresight because they suggest that providing children with specific cues will also facilitate the construction of a future event. Accordingly, one would predict that the presence of cues in the current context might facilitate episodic foresight in younger children. However, only older children would be able to generate the necessary cues to think about a future event. This is an issue that requires additional empirical attention.

#### Non-verbal tasks

In this section, we distinguish “item-choice” tasks used by Atance and Meltzoff (2005) and Russell et al. (2010), from those tasks that have used a “two-rooms” methodology (e.g., Suddendorf et al., 2011; Atance and Sommerville, 2014). Recall that Atance and Meltzoff (2005) found that 3-, 4-, and 5-year-olds were all significantly above chance in selecting a correct item to address a potential future physiological state (e.g., Band-Aids in case they get hurt). In addition to this more “non-verbal” item choice measure, children were also asked to explain why they selected the item that they did (e.g., “How come you chose the raincoat?”). Here, age differences were detected, with both 4- and 5-year-olds providing significantly more justifications that referenced a future physiological state (e.g., “I might get wet”) than did 3-year-olds. Clearly, having some knowledge (likely semantic in this case) about the fact that raincoats are needed for rain or that Band-Aids are needed when one gets hurt is necessary for task success. In fact, this may be why 3-year-olds did so well on the item-choice measure.

However, is this semantic knowledge sufficient? The fact that there were nonetheless significant age differences even on the item-choice measure suggests that it was not. Could episodic memory contributions be important as well? For example, is it possible that accessing a past episode of having gotten wet because of not having a raincoat increases children’s odds of selecting the correct item? And, might episodic memory (and a better sense of the “subjective” self, in particular) explain why older children were much more likely than younger children to actually link their choices to the self by formulating such future-oriented justifications as “*I* might get wet”?

Along the same lines, in Russell et al.’s (2010) study, only 5-year-olds successfully chose the correct items (i.e., straw and box) for a game of blow football “tomorrow.” In contrast, when faced with a decision about a game of blow football “right now,” even 3- and 4-year-olds were significantly above chance. This suggests that their failure in the future/tomorrow condition cannot be attributed to lacking the semantic knowledge necessary to solve the task. Accordingly, it is difficult to argue that the 5-year-olds passed the future version of the task by simply drawing on semantic memory (otherwise, younger children should also have passed). Rather, it is possible that the younger children correctly chose the items in the present condition because they did not need to rely on episodic memory. In contrast, thinking about what would be needed in their own *future* (i.e., drawing on the episodic system) may have overly taxed their planning skills. Interestingly, 4-year-olds had less difficulty choosing the correct items that another child might need in the future than they did choosing the items that they, themselves, would need. This supports the claim that imagining one’s *own future* might rely more heavily on episodic processes than imagining someone else’s future. Such self-other differences may in fact shed light on the extent to which semantic and episodic memory processes are involved in episodic foresight. More broadly, the role that such abilities as theory of mind play in episodic foresight (e.g., Suddendorf and Corballis, 2007) are important to consider because, oftentimes, thinking about one’s future self – a self that may have different desires, thoughts, etc. – is similar to thinking about another person. In doing so, it may also be possible to isolate the processes that are unique to adopting one’s own future perspective (and, hence, “episodic foresight”) vs. the perspective of another.

We move next to tasks that have used two-room methodologies (e.g., Suddendorf et al., 2011; Atance and Sommerville, 2014) and, more specifically, the role that both episodic and semantic memory may play in task success. Recall that in one task used by Atance and Sommerville (2014), children were presented with a glass of juice glued to a tray (and no straw with which to drink it) in one room; several minutes later, in another room, they were given various items with which to solve the problem. In this case, the correct choice was a straw. Arguably, at the most basic level, children needed to remember that there was a glass of juice on the table, for example. If children cannot remember this information, then this precludes them from choosing the correct item with which to solve the problem at a later time (and, hence, making the correct “future-oriented” choice). Interestingly, in Suddendorf et al.’s (2011) control condition (i.e., no delay, same/different spatial location) both younger and older children chose the correct tool with which to solve the problem (in this case, the correctly shaped key to open a locked box). This implies that younger children’s planning skills are already present. However, when children also needed to rely on their memory of the past event, these planning skills were insufficient to succeed. This interpretation is consistent with Hayne et al.’s (2011) claim that 3-year-olds form episodic memories but these memories fade with time (see also Scarf et al., 2013).

However, are there other aspects of the child’s past experience that could also play a role in task success? Note that, at least in Suddendorf et al.’s (2011) study, the “associative” semantic argument (e.g., keys are used to open boxes) does not apply. This is because, in this study, children were presented with several keys and they could not merely succeed by making an association between “key” and “box.” Therefore, it seems more plausible to argue that children needed to remember specific information about which key was needed (though it is important to note that retrieving information about the key does not necessarily entail remembering the event in which the key was first encountered, Martin-Ordas et al., 2012).

The idea that, in some tasks, it may be possible to isolate a specific contribution of episodic memory is also nicely illustrated in a study by Metcalf and Atance (2011). These authors used a “saving marbles” task in which 3-, 4-, and 5-year-olds were shown two marble games in two different rooms. In one condition, a small/less-desirable marble run was in the first room and a large/more-desirable one was in the second. Children were given a limited number of marbles and were told that they would spend 3 min in the first room and then, afterwards, 3 min in the second room. However, children were also familiarized with both marble runs such that the experimenter showed them that when they put a marble down the run they were unable to retrieve it again. Of interest was whether children would save marbles for the second room, rather than use them all up in the first. Interestingly, children saved very few marbles and performance did not differ as a function of age.

However, children also received a (surprise) second trial of the “marble game” to see if they could learn from their past experience (e.g., disappointment at having very few/no marbles left to use in the large marble run). Interestingly, children saved significantly more marbles in this second trial and, again, there were no age-related differences. An interesting way to interpret these results is to argue that children’s “semantic” knowledge of marble runs, and the fact that marbles go down marble runs, likely did not change during the course of the study. However, the one-time event (i.e., trial 1) of not having marbles to use in the large marble run then appeared to influence children’s saving behavior. Thus, one might conclude that episodic memory was contributing to the increase in marble saving in this trial. The fact that trial 2 immediately followed trial 1, and that children may have been quite disappointed at not having any (or few) marbles to use in the large marble run may explain why the amount of marbles saved increased across all three age groups, and not just for the older children. Conversely, it is possible that because the task involved more than one trial, children may have learned after the first trial that saving is a good strategy – such a rule being a function of memory that is more semantic, rather than episodic, in nature.

When is it necessary, then, to remember emotional aspects of the episode itself (e.g., disappointment at not having a straw to drink the juice, put a marble in the marble run, etc.), and not just the physical features of the problem (e.g., that the glass is stuck to the tray)? In those instances in which the materials in question are ones that are familiar to children (e.g., a glass of juice and a straw), then surely knowledge about these materials and possible associations between them (e.g., straws are associated with glasses, are used to drink, etc.) would certainly facilitate the activation of the correct future-oriented response when the straw is seen amongst a number of distracter items. However, this association would not work when the future event involves the use of a straw but for a different problem (e.g., to blow a ball). Thus, remembering a situation in which I used a straw for a different purpose than to drink might be necessary for successful performance in Russell et al. (2010), for example. These experiences could be considered more episodic in nature because they would likely constitute a one-time event in the past that children draw upon to help their future planning. This would be different from having the general knowledge that “straws are for drinks.” Taking this a step further, a child who has both well-developed “semantic” memories (e.g., straws are for drinks) and “episodic” memories (e.g., a personally relevant past instance in which they used a straw for a different purpose) would clearly be at an advantage in this task.

More specifically, to solve an item-choice task, children may first draw upon some sort of knowledge/semantic information that forms the basis for guiding a future action. In some cases, most notably when the problem in question is more associative (e.g., straws go with juice), there may not be any other information/memories that need to be drawn upon to succeed in selecting the correct item. However, perhaps in contexts in which an associative link is more difficult to make, accessing a specific episode in one’s past that is pertinent to the problem/situation in question would also benefit the child. Whereas the former would be more objective/semantic knowledge, the latter would be more subjective/episodic. This idea is intimately related to the flexibility criterion proposed by Suddendorf and Corballis (2007). We suggest that the more novel the combination of past events used to construct the future event, the more flexible and creative (or future-oriented) the thought of an event becomes (Osvath and Martin-Ordas, in press).

So far, we have been describing tasks of episodic foresight and we have argued that both semantic and episodic memory likely play a role in task success. We have also argued that, in some instances, having the relevant semantic information may be sufficient to succeed on an episodic foresight/planning task. As such, an important question to ask is what episodic memory adds to an individual’s ability to mentally project into the future. Although the notion of “flexibility” is argued to be critical (Suddendorf and Corballis, 2007), empirically addressing this claim is challenging. We have also identified ways in which episodic memory may facilitate children’s performance on a variety of episodic foresight tasks. However, we discuss this particular issue in more detail in this next section and also suggest some directions for future research.

## THE ROLE OF MEMORY IN EPISODIC FORESIGHT: DIRECTIONS FOR FUTURE RESEARCH

There is no doubt that some form of memory is involved in the developmental tasks that have been used to assess episodic foresight. However, one of the limitations of these tasks is that they were not designed to directly assess the roles of episodic and semantic memory in children’s performance. Therefore, future research should move beyond assessing correlations between children’s memory for the past and thought about the future and empirically address the extent to which episodic and semantic memory contribute to task success. We touched on several ways to do so throughout our paper, including examining potential self-other differences in episodic foresight, as well as the idea of holding either the semantic or episodic memory demands of a task constant and then observe how this impacts children’s performance. For example, we argued that in Metcalf and Atance’s (2011) saving task, improvements on the second trial may have been due to children’s ability to mentally re-experience the disappointment (i.e., episodic memory) of having few/no marbles to put into the large marble run, and not due to any new semantic knowledge about marble runs. In what follows we further elaborate on different ways to study how both semantic and episodic memory contribute to episodic foresight.

### GENERAL VS. UNIQUE EVENTS/GENERATIVE VS. DIRECT GENERATION OF FUTURE EVENTS

Suddendorf and Corballis (2007) and Suddendorf et al. (2011) have argued that an important criterion of episodic foresight is that it be based on a single exposure to an event. Two important issues can be raised with respect to this criterion. First, most memory systems require repeated exposure to a stimulus for it to be retained (Morris, 2001). Moreover, certain conditioning procedures have demonstrated that single trial exposure does not necessarily rule out associative learning (e.g., Garcia and Koelling, 1966). Second, we have already mentioned that some of our memories do not necessarily involve *unique* events (e.g., my first conference talk) but, rather, *general* events (e.g., memories from the time I was doing my Ph.D). Yet, the “single exposure” criterion would necessarily preclude such general events from being considered as critical for episodic foresight. As such, we argue that an important direction for future research is to analyze the roles of both unique and general events to episodic foresight. One way to do so is by asking parents to report general (e.g., swimming lessons) and unique (e.g., going to Disney World) events from their child’s past. Then, children could be asked to imagine and describe either a general future event (e.g., talk about future tennis lessons) based on a general memory (e.g., past swimming lessons) or to describe a unique future event (e.g., future trip to Disneyland) that is related to a unique past event (e.g., past trip to Disney World). Because memories for general events are more semantic than episodic in nature, one would expect younger children to succeed at constructing and talking about general future events (e.g., tennis lessons) that are based on general past events (e.g., swimming lessons). In contrast, younger children would be expected to have more difficulty constructing unique future events (e.g., trip to Disneyland) when they can only rely on unique past events (e.g., past trip to Disney World). In contrast, older children should be able to use information from both general and unique events. Therefore, one would expect them to successfully describe future events based on both general and unique events.

Earlier in our paper, we argued that the studies by Hayne et al. (2011) and Busby and Suddendorf (2005) could be re-interpreted along the lines of generative vs. direct retrieval (Conway, 2005). Note, though, that these studies did not directly assess this issue and, hence, more controlled experiments are needed. Therefore, one could extend the line of research suggested above by asking children to use this information (e.g., semantic and episodic memories) to create a specific future event (e.g., talk about your swimming lesson tomorrow) or to imagine what they will do in the future (e.g., what are you going to do tomorrow?). This would allow us to directly test the idea of *generative* (i.e., elaborating upon one’s own cue/event to think about a future event) vs. *direct* (i.e., the specific cue/event is provided by the experimenter) generation of future events. If providing children with a more specific cue helps them to more accurately describe a future event, then we would expect older children to perform better than younger children when no specific cue about a future event is provided. This is because while younger children might be able to elaborate upon an event that is provided to them (as in Hayne et al., 2011), older children might be able to think about the events that could happen to them even when they are asked to generate them on their own (e.g., what are you going to do tomorrow?).

### FLEXIBILITY

Imagining a future event in a constructive manner (i.e., being able to interchange and combine features of past events in order to imagine new ones) is arguably highly adaptive (Schacter and Addis, 2007; Suddendorf and Corballis, 2007). It follows, then, that flexibility “frees” us from the current spatio-temporal context and allows us to use past experience to imagine future situations (e.g., solving problems) that we have not previously experienced. However, semantic learning also provides us with flexible behaviors (Osvath and Martin-Ordas, in press). Indeed, we can apply a skill (or objects) to new problems that are different from the problems that served as the basis for training/learning (e.g., stimulus generalization). For example, we can use a pen to write with, but we can also use it as a blowpipe. We can do this solely by having an understanding of the function and properties of pens. In other words, imagining how to use a pen as a blowpipe does not rest on our ability to remember a *specific* event in which we needed a blowpipe. If existing semantic knowledge suffices for future-oriented decisions, when does the use of episodic projection become necessary?

One possibility is that the more novel the combinations of past events, the more creative a behavior/response becomes. Imagine the following scenario: You know that tools are always kept in the toolbox; however, in one past instance you placed the screwdriver in a different location (e.g., inside a drawer). If you now needed to find the screwdriver, where would you search for it? If relying solely on your semantic knowledge, you would go and search inside the toolbox because that is where the screwdriver usually is. But what would you do if you did not find it there? In this case, only the memory of the specific event in which you put the screwdriver inside the drawer would allow you to successfully search for it in the drawer (and not in the cupboard). We suggest that developing experimental tasks that reflect this example could provide insights about the specific contributions of episodic and semantic memory in episodic foresight – especially in young children. More specifically, we would predict that, in such scenarios, children with a non-fully developed episodic system would have difficulty. In contrast, older children would be able to retrieve the unique past experience needed to successfully find the tool.

In summary, we have described different experimental approaches that have been used to study young children’s episodic foresight. Developmental findings suggest that the capacity for episodic foresight develops substantially between the ages of 3 and 5. We have pointed out that even though episodic memory seems to be intimately related to episodic foresight, the role that both semantic and episodic memory play in episodic foresight is only now starting to be addressed. We also suggest that episodic foresight might be possible without the need to recall episodic memories and, based on this idea, we have proposed new avenues of research that could provide us with important insights about the ontogeny of episodic foresight.

## Conflict of Interest Statement

The authors declare that the research was conducted in the absence of any commercial or financial relationships that could be construed as a potential conflict of interest.

## References

[B1] Addis D. R. Sacchetti D. C. Ally B. A. Budson A. E. Schacter D. L. (2009). Episodic simulation of future events is impaired in mild Alzheimer’s disease. *Neuropsychologia* 47 2660–2671 10.1016/j.neuropsychologia.2009.05.018 19497331 PMC2734895

[B2] Addis D. R. Wong A. T. Schacter D. L. (2007). Remembering the past and imagining the future: common and distinct neural substrates during event construction and elaboration. *Neuropsychologia* 45 1363–1377 10.1016/j.neuropsychologia.2006.10.016 17126370 PMC1894691

[B3] Atance C. M. (2008). “From the past into the future: the developmental origins and trajectory of episodic future thinking,” in *Handbook of Behavioral Neuroscience: Episodic Memory Research* eds Dere E. Easton A. Nadel L. Huston J. P. (Amsterdam: Elsevier Press) 99–114

[B4] Atance C. M. Martin-Ordas G. (2014). “Projecting the self into the future,” in *Wiley-Blackwell Handbook on the Development of Children’s Memory* eds Bauer P. J. Fivush R. (Malden, MA: John Wiley & Sons Ltd.) 645–664

[B5] Atance C. M. Meltzoff A. N. (2005). My future self: young children’s ability to anticipate and explain future states. *Cogn. Dev.* 20 341–361 10.1016/j.cogdev.2005.05.001 23956493 PMC3744374

[B6] Atance C. M O’Neill D. K. (2001). Episodic future thinking. *Trends Cogn. Sci.* 5 533–539 10.1016/S1364-6613(00)01804-0 11728911

[B7] Atance C. M O’Neill D. K. (2005). The emergence of episodic future thinking in humans. *Learn. Motiv.* 36 126–144 10.1016/j.lmot.2005.02.003

[B8] Atance C. M. Sommerville J. A. (2014). Assessing the role of memory in preschoolers’ performance on episodic foresight tasks. *Memory* 22 118–128 10.1080/09658211.2013.820324 23889532

[B9] Balota D. A. Coane J. H. (2008). “Semantic memory,” in *Handbook of Learning and Memory: A Comprehensive Reference* eds Byrne J. H. Eichenbaum H. Menzel R. Roediger H. L. III Sweatt D. (Amsterdam: Elsevier) 512–531

[B10] Barsalou L. W. (1988). “The content and organization of autobiographical memories,” in *Remembering Reconsidered: Ecological and Traditional Approaches to the Study of Memory* eds Neisser U. Winograd E. (New York, NY: Cambridge University Press) 193–243

[B11] Buckner R. L. Carroll D. C. (2007). Self-projection and the brain. *Trends Cogn. Sci.* 11 49–57 10.1016/j.tics.2006.11.004 17188554

[B12] Busby J. Suddendorf T. (2005). Recalling yesterday and predicting tomorrow. *Cogn. Dev.* 20 362–372 10.1016/j.cogdev.2005.05.002

[B13] Chalfonte B. L. Johnson M. K. (1996). Feature memory and binding in young and older adults. *Mem. Cogn.* 24 403–416 10.3758/BF03200930 8757490

[B14] Cheke L. G. Clayton N. S. (2010). Mental Time Travel in Animals. *Wiley Interdiscip. Rev. Cogn. Sci.* 1 915–930 10.1002/wcs.59 26271786

[B15] Conway M. A. (2001). Sensory-perceptual episodic memory and its context: autobiographical memory. *Philos. Trans. R. Soc. Lond. Biol. Sci.* 356 1375–1384 10.1098/rstb.2001.0940 11571029 PMC1088521

[B16] Conway M. A. (2005). Memory and the self. *J. Mem. Lang.* 52 594–628 10.1016/j.jml.2005.08.005

[B17] Conway M. A. Fthenaki A. (2000). “Disruption and loss of autobiographical memory,” in *Handbook of Neuropsychology: Memory and Its Disorders* 2nd Edn eds Boller F. Grafman (Amsterdam: Elsevier Science B.V.) 281–312

[B18] D’Argembeau A. Renaud O Van der Linden M. (2011). Frequency, characteristics, and functions of future-oriented thoughts in daily life. *Appl. Cogn. Psychol.* 35 96–103 10.1002/acp.1647

[B19] Dudai Y. Carruthers M. (2005). The Janus face of Mnemosyne. *Nature* 434 567 10.1038/434567a 15800602

[B20] Duval C. Desgranges B. De La Sayette V. Belliard S. Eustache F. Piolino P. (2012). What happens to personal identity when semantic knowledge degrades? A study of the self and autobiographical memory in semantic dementia. *Neuropsychologia* 50 254–256 10.1016/j.neuropsychologia.2011.11.019 22155259

[B21] Eichenbaum H. (1997). How does the brain organize memories? *Science* 277 330–332 10.1126/science.277.5324.330 9518364

[B22] Eisenberg A. R. (1985). Learning to describe past experience in conversation. *Discourse Process.* 8 177–204 10.1080/01638538509544613

[B23] Friedman W. J. (1990). *About Time: Inventing the Fourth Dimension*. Cambridge, MA: MIT Press

[B24] Gadian D. G. Aicardi J. Watkins K. E. Porter D. A. Mishkin M. Vargha-Khadem F. (2000). Developmental amnesia associated with early hypoxic–ischaemic injury. *Brain* 123 499–507 10.1093/brain/123.3.499 10686173

[B25] Gamboz N. De Vito S. Bradimonte M. A. Pappalardo S. G. Galeone F. Iavarone A. (2010). Episodic future thinking in amnesic mild cognitive impairment. *Neuropsychologia* 48 2091–2097 10.1016/j.neuropsychologia.2010.03.030 20381507

[B26] Garcia J. Koelling R. A. (1966). Relation of cue to consequence in avoidance learning. *Psychonom. Sci.* 4 123–124 10.3758/BF03342209

[B27] Gilbert D. T. Wilson T. D. (2007). Prospection: experiencing the future. *Science* 317 1351–1354 10.1126/science.1144161 17823345

[B28] Graham K. S. Lee A. C. H. Brett M. Patterson K. (2003). The neural basis of autobiographical and semantic memory: new evidence from three PET studies. *Cogn. Affect. Behav. Neurosci.* 3 234–254 10.3758/CABN.3.3.234 14672158

[B29] Graham K. S. Simons J. S. Pratt K. H. Patterson K. Hodges J. R. (2000). Insights from semantic dementia on the relationship between episodic and semantic memory. *Neuropsychologia* 38 313–324 10.1016/S0028-3932(99)00073-1 10678697

[B30] Greenberg D. L. Verfaellie M. (2010). Interdependence of episodic and semantic memory: evidence from neuropsychology. *J. Int. Neuropsychol. Soc.* 16 748–753 10.1017/S1355617710000676 20561378 PMC2952732

[B31] Harner L. (1982) “Talking about the past and the future,” in *The Developmental Psychology of Time* ed. Friedman W. J. (New York, NY: Academic Press) 141–170

[B32] Hassabis D. Kumaran D. Vann S. D. Maguire E. A. (2007). Patients with hippocampal amnesia cannot imagine new experiences. *Proc. Natl. Acad. Sci. U.S.A.* 104 1726–1731 10.1073/pnas.0610561104 17229836 PMC1773058

[B33] Hayne H. Gross J. Mcnamee S. Fitzgibbon O. Tustin K. (2011). Episodic memory and episodic foresight in 3-and 5-year-old children. *Cogn. Dev.* 26 343–355 10.1016/j.cogdev.2011.09.006

[B34] Hirano M. Noguchi K. (1998). Dissociation between specific personal episodes and other aspects of remote memory in a patient with hippocampal amnesia. *Percept. Mot. Skills* 87 99–107 10.2466/pms.1998.87.1.99 9760633

[B35] Hudson J. A. Shapiro L. R. Sosa B. B. (1995). Planning in the real world: preschool children’s scripts and plans for familiar events. *Child Dev.* 66 984–998 10.2307/1131793 7671660

[B36] Irish M. Addis D. R. Hodges J. R. Piguet O. (2012). Considering the role of semantic memory in episodic future thinking: evidence from semantic dementia. *Brain* 135 2178–2191 10.1093/brain/aws119 22614246

[B37] Irish M. Piguet O. (2013). The pivotal role of semantic memory in remembering the past and imagining the future. *Front. Behav. Neurosci.* 7:27. 10.3389/fnbeh.2013.00027 PMC3615221 23565081

[B38] Kapur N. (1999). Syndromes of retrograde amnesia: a conceptual and empirical synthesis. *Psychol. Bull.* 125 800–825 10.1037/0033-2909.125.6.800 10589303

[B39] Klein S. B. (2013). Making the case that episodic recollection is attributable to operations occurring at retrieval rather than to content stored in a dedicated subsystem of long-term memory. *Front. Behav. Neurosci.* 7:3 10.3389/fnbeh.2013.00003 PMC3561741 23378832

[B40] Klein S. B. Loftus J. Kihlstrom J. F. (2002). Memory and temporal experience: The effects of episodic memory loss on an amnesic patient’s ability to remember the past and imagine the future. *Soc. Cogn.* 20 353–379 10.1521/soco.20.5.353.21125

[B41] Kwan D. Carson N. Addis D. R. Rosenbaum R. S. (2010). Deficits in past remembering extend to future imagining in a case of developmental amnesia. *Neuropsychologia* 48 3179–3186 10.1016/j.neuropsychologia.2010.06.011 20561535

[B42] Kwan D. Craver C. F. Green L. Myerson J. Rosembaum R. S. (2013). Dissociations in future thinking following hippocampal damage: evidence from discounting and time perspective in episodic amnesia. *J. Exp. Psychol. Gen.* 142 1355–1369 10.1037/a0034001 23978187

[B43] Maguire E. A. Vargha-Khadem F. Hassabis D. (2010). Imagining fictitious and future experiences: evidence from developmental amnesia. *Neuropsychologia* 48 3187–3192 10.1016/j.neuropsychologia.2010.06.037 20603137 PMC2937213

[B44] Martin-Ordas G. Atance C. M. Louw A. (2012). The role of episodic and semantic memory in episodic foresight. *Learn. Motiv*. 43 209–219 10.1016/j.lmot.2012.05.011

[B45] McKinnon M. C. Black S. E. Miller B. Moscovitch M. Levine B. (2006). Autobiographical memory in semantic dementia: implications for theories of limbic-neocortical interaction in remote memory. *Neuropsychologia* 44 2421–2429 10.1016/j.neuropsychologia.2006.04.010 16765999 PMC1995668

[B46] Metcalf J. L. Atance C. M. (2011). Do preschoolers save to benefit their future selves? *Cogn. Dev.* 26 371–382 10.1016/j.cogdev.2011.09.003

[B47] Morris R. G. M. (2001). Episodic-like memory in animals: psychological criteria, neural mechanisms and the value of episodic-like tasks to investigate animal models of neurodegenerative disease. *Phil. Trans. R. Soc. Lond. B* 356 1453–1465 10.1098/rstb.2001.0945 11571036 PMC1088528

[B48] Neisser U. (1981). John Dean’s memory: a case study. *Cognition* 9 1–22 10.1016/0010-0277(81)90011-1 7196816

[B49] Nelson K. (1989). *Narratives from the Crib*. Cambridge, MA: Harvard University Press

[B50] Newcombe N. S. Lloyd M. E. Ratliff K. R. (2007). “Development of episodic and autobiographical memory: a cognitive neuroscience perspective,” in *Advances in Child Development and Behavior* Vol. 35 ed. Kail R. V. (San Diego, CA: Elsevier) 37–85 10.1016/b978-0-12-009735-7.50007-4 17682323

[B51] Okuda J. Fujii T. Ohtake H. Tsukiura T. Tanji K. Suzuki K. (2003). Thinking of the future and past: The roles of the frontal pole and the medial temporal lobes. *Neuroimage* 19 1369–1380 10.1016/S1053-8119(03)00179-4 12948695

[B52] Osvath M. Martin-Ordas G. (in press). The future of future-oriented cognition in non-humans: theory, and the empirical case of the great apes. *Philos. Trans. R. Soc. Lond. B Biol. Sci.* 10.1098/rstb.2013.0486 PMC4186238 25267827

[B53] Pillemer D. (2003). Directive functions of autobiographical memory: the guiding power of specific episodes. *Memory* 11 193–202 10.1080/741938208 12820831

[B54] Quon E. Atance C. M. (2010). A comparison of preschoolers’ memory, knowledge, and anticipation of events. *J. Cogn. Dev.* 11 37–60 10.1080/15248370903453576

[B55] Race E. Keane M. M. Verfael-lie M. (2011). Medial temporal lobe damage causes deficits in episodic memory and episodic memory and episodic future thinking not attributable to deficits in narrative construction. *J. Neurosci.* 31 10262–10269 10.1523/JNEUROSCI.1145-11.2011 21753003 PMC4539132

[B56] Redshaw J. Suddendorf T. (2013). Foresight beyond the very next event: four-year-olds can link past and deferred future episodes. *Front. Psychol.* 4:404. 10.3389/fpsyg.2013.00404 PMC3705196 23847575

[B57] Russell J. Alexis D. Clayton N. S. (2010). Episodic future thinking in 3–5 year-old children: the ability to thinking of what will be needed from a different point of view. *Cognition* 114 56–71 10.1016/j.cognition.2009.08.013 19781693

[B58] Sachs J. (1983). “Talking about the there and then: the emergence of displaced reference in parent–child discourse,” in *Children’s Language* ed. Nelson K. Vol. 4 (Hillsdale, NJ: Lawrence Erlbaum Associates) 1–28

[B59] Scarf D. Gross J. Colombo M. Hayne H. (2013). To have and to hold: episodic memory in 3- and 4-year-old children. *Dev. Psychobiol.* 55 125–132 10.1002/dev.21004 22213009

[B60] Schacter D. L. Addis D. R. (2007). The ghosts of past and future. *Nature* 445:27 10.1038/445027a 17203045

[B61] Schacter D. L. Addis D. R. Buckner R. L. (2007). The prospective brain: remembering the past to imagine the future. *Nat. Rev. Neurosci.* 8 657–661 10.1038/nrn2213 17700624

[B62] Schacter D. L. Addis D. R. Buckner R. L. (2008). Episodic simulation of future events: concepts, data, and applications. *Ann. N. Y. Acad. Sci.* 1124 39–60 10.1196/annals.1440.001 18400923

[B63] Snowden J. Griffiths H. Neary D. (1994). Semantic dementia: autobiographical contribution to preservation of meaning. *Cogn. Neuropsychol.* 11 265–288 10.1080/02643299408251976

[B64] Squire L. R. Zola S. M. (1998). Episodic memory, semantic memory and amnesia. *Hippocampus* 8 205–211 10.1002/(SICI)1098-1063(1998)8:3<205::AID-HIPO3>3.0.CO;2-I 9662135

[B65] Squire L. R. Stark C. E. L. Clark R. E. (2004). The medial temporal lobe. *Annu. Rev. Neurosci.* 27 279–306 10.1146/annurev.neuro.27.070203.144130 15217334

[B66] Suddendorf T. (2010). Linking yesterday and tomorrow: preschoolers’ ability to report temporally displaced events. *Br. J. Dev. Psychol.* 28 461–498 10.1348/026151009X479169 20481400

[B67] Suddendorf T. Busby J. (2005). Making decisions with the future in mind: developmental and comparative identification of mental time travel. *Learn. Motiv.* 36 110–125 10.1016/j.lmot.2005.02.010

[B68] Suddendorf T. Corballis M. C. (1997). Mental time travel and the evolution of the human mind. *Genet. Soc. Gen. Psychol. Monogr.* 123 133–167 10.1098/rstb.2008.0301 9204544

[B69] Suddendorf T. Corballis M. C. (2007). The evolution of foresight: what is mental time travel and is it unique to humans? *Behav. Brain Sci.* 30 299–313 10.1017/S0140525X07001975 17963565

[B70] Suddendorf T. Nielsen M von Gehlen R. (2011). Children’s capacity to remember a novel problem and to secure its future solution. *Dev. Sci.* 14 26–33 10.1111/j.1467-7687.2010.00950 21159085

[B71] Szpunar K. K. (2010). Episodic future thought: an emerging concept. *Perspect. Psychol. Sci.* 5 142–162 10.1177/1745691610362350 26162121

[B72] Szpunar K. K. McDermott K. B. (2008). Episodic future thought and its relation to remembering: evidence from ratings of subjective experience. *Conscious. Cogn.* 17 330–334 10.1016/j.concog.2007.04.006 17540581

[B73] Szpunar K. K. Watson J. M. McDermott K. B. (2007). Neural substrates of envisioning the future. *Proc. Natl. Acad. Sci. U.S.A.* 104 642–647 10.1073/pnas.0610082104 17202254 PMC1761910

[B74] Tulving E. (1972). “Episodic and semantic memory,” in *Organization of Memory* eds Tulving E. Donaldson W. (New York, NY: Academic Press Inc.) 381–403

[B75] Tulving E. (1983). *Elements of Episodic Memory*. Oxford: Clarendon Press

[B76] Tulving E. (1985). Memory and consciousness. *Can. Psychol.* 26 1–12 10.1037/h0080017

[B77] Tulving E. (1995). “Organization of memory: Quo Vadis?,” in *The Cognitive Neuroscience* ed. Gazzaniga M. S. (Cambridge, MA: MIT Press) 839–847

[B78] Tulving E. (2005). “Episodic memory and autonoesis: uniquely human?,” in *The Missing Link in Cognition* eds Terrace H. S Metcalfe J. (Oxford: Oxford University Press) 3–56

[B79] Vargha-Khadem F. Gadian D. G. Watkins K. E. Connelly A. Van Paesschen W. Mishkin M. (1997). Differential effects of early hippocampal pathology on episodic and semantic memory. *Science* 277 376–380 10.1126/science.277.5324.376 9219696

[B80] Weist R. M. (1989). “Time concepts in language and thought: filling the Piagetian void from two to five years,” in *Time and Human Cognition: A Life-Span Perspective* eds Levin I. Zakay D. (Amsterdam: Elsevier) 63–118 10.1016/S0166-4115(08)61039-0

[B81] Wheeler M. A. McMillan C. T. (2001). Focal retrograde amnesia and the episodic-semantic distinction. *Cogn. Affect. Behav. Neurosci.* 1 22–36 10.3758/CABN.1.1.22 12467101

[B82] Wheeler M. A. Stuss D. T. Tulving E. (1997). Toward a theory of episodic memory: the frontal lobes and autonoetic consciousness. *Psychol. Bull.* 121 331–354 10.1037/0033-2909.121.3.331 9136640

[B83] Williams J. M. G. Ellis N. C. Tyers C. Healy H. Rose G. MacLeod A. K. (1996). The specificity of autobiographical memory and imageability of the future. *Mem. Cogn.* 24 116–25 10.3758/BF03197278 8822164

